# Transcranial direct current stimulation in affecting neuropsychiatric symptoms of post-COVID syndrome: No change in microstates and functional connectivity

**DOI:** 10.1371/journal.pone.0351407

**Published:** 2026-06-26

**Authors:** Nina Biačková, Olga Laskov, Andrea Adamová, Václava Piorecká, Jan Štrobl, Stanislav Jiříček, Jan Hubený, Vlastimil Koudelka, Anna Čechová, Natalie-Anna Böhmová, Monika Klírová

**Affiliations:** 1 National Institute of Mental Health, Klecany, Czech Republic; 2 Third Faculty of Medicine, Charles University, Prague, Czech Republic; 3 Department of Biomedical Technology, Faculty of Biomedical Engineering, Czech Technical University, Prague, Czech Republic; 4 Department of Cybernetics, Faculty of Electrical Engineering, Czech Technical University, Prague, Czech Republic; 5 Department of Complex Systems, Institute of Computer Science of the Czech Academy of Sciences, Prague, Czech Republic; University of Minho: Universidade do Minho, PORTUGAL

## Abstract

**Introduction:**

Neuropsychiatric symptoms of post-covid syndrome (PCS) involve fatigue, brain fog, cognitive impairment, or symptoms of depression and anxiety. These symptoms are in other diagnoses associated with electroencephalography (EEG) changes in microstates and functional connectivity. Transcranial direct current stimulation (tDCS) is effective in alleviating certain abovementioned symptoms in other diagnoses. The main objective was to evaluate EEG changes in PCS patients after tDCS intervention.

**Methods:**

A randomised control trial (registered in ISRCTN database, No.: ISRCTN10942585) with 35 participants (n = 33 received the intervention) involved 20 applications (over four weeks) of tDCS (anode over F3, cathode over F4, duration 30 min, intensity 2mA). Participants were divided into active (n = 16) and sham (n = 17) groups. Resting-state EEG was measured at baseline, after two weeks (T1), and after the intervention (four weeks, T2) as an exploratory endpoint. Microstates and functional connectivity between F3 and F4 were analysed; differences between active and sham groups in change from baseline to T1 and from baseline to T2 were evaluated.

**Results:**

EEG measurements from 26 participants (n = 13 for active and n = 13 for sham group) were included into the statistical analysis. No significant results were found. No serious adverse events were noted.

**Conclusions:**

We did not find a significant difference in selected EEG parameters between active and sham groups. The possible reasons include choice of tDCS parameters (electrode montage or stimulation duration), and EEG variables selected for evaluation (microstates and functional connectivity). These results are in line with the results of cognitive and clinical assessment. However, the EEG endpoints were exploratory and the study was primarily powered for clinical results, therefore small effects cannot be excluded due to limited statistical power.

## Introduction

Post-covid syndrome (PCS; also known as post-acute sequelae of SARS-CoV-2 infection – PASC), i.e., symptoms prevailing for minimum of one month [1–3] after acute Covid-19 infection, may affect up to 40% of infected patients [4]. Out of these symptoms, the neuropsychiatric subgroup includes fatigue with ca. 37%, brain fog with 32% [5], and cognitive impairment with 22% prevalence [6]. In the psychiatric subgroup, symptoms of depression and anxiety are present in 12% and 23% of patients [5]. Pathophysiological mechanisms that may contribute to this condition include hypometabolism, autoimmune encephalitis [7] and general inflammatory state [8], endothelial dysfunction and myelin loss [8,9]. In imaging studies, changes in functional connectivity, and hypoconnectivity between left and right parahippocampal areas, orbitofrontal, and cerebellar areas have been found [10]. Among possible treatment options are corticoids and intravenous immunoglobulin application [11], physical rehabilitation [12], cognitive training [13], and oxygen therapy [14]. Antidepressants, such as selective serotonin reuptake inhibitors (SSRIs) may be used in case of depressive symptoms [15].

Besides these options, interventions from the non-invasive brain stimulation field are also under investigation. Particularly, the use of transcranial direct current stimulation (tDCS) suggests a promising approach. TDCS is effective in depression, but also in neurological and neuropsychiatric disorders, such as chronic pain, or fatigue in fibromyalgia [16]. Certain aspects of cognition might be improved by tDCS [17], for example, in depression, which is besides affective impairment also characterized by disturbance of cognitive function, tDCS was found to improve cognitive control [18]. Furthermore, a meta-analysis [19] found an enhancement of working memory after tDCS treatment across multiple disorders, including depression, dementia, or schizophrenia. TDCS changes cortical excitability (increase in anodal tDCS, decrease in cathodal tDCS) [20] and promotes synaptic plasticity [21], although here, more studies with bio- or imaging markers are needed. In the first years after the Covid-19 outbreak, case studies applying tDCS emerged. An improvement in fatigue, and in certain cognitive domains, such as attention, processing speed, or cognitive flexibility, was found [2,22]. These first results, as well as effectivity of tDCS in other diagnoses, were the basis of applying tDCS in neuropsychiatric symptoms of PCS. Therefore, the aim of this clinical trial was to test the efficacy of tDCS intervention on the clinical and cognitive status of neuropsychiatric PCS patients. No significant differences were found between the active and sham groups; results are described in [23]. A recent meta-analysis, published after the completion of this study, describes an improvement of PCS related fatigue, but no improvements in cognition after tDCS [24].

However, at the time of realisation of this study, there was no previous randomised control trial (RCT), which also measured electrophysiological changes through electroencephalography (EEG). The choice of evaluated parameters was based on the then-available information on EEG changes in PCS patients, and on EEG findings from other diagnoses. Changes in microstates (MS), i.e., quasi-stable states observed in EEG, specifically in the duration and frequency parameters were described [25]. MS are also changed in diagnoses with symptoms similar to PCS, such as depression [26], and also in fatigue in multiple sclerosis [27], or fibromyalgia [28]. Moreover, it seems that tDCS is able to change microstate maps in patients with depression [29], schizophrenia [30], and obsessive-compulsive disorder, where it is also connected with an improvement of their clinical status [31]. TDCS may also influence network synchronization and functional connectivity, either directly around the electrodes [32], as well as in related networks, e.g., fronto-parietal network in patients with anxiety and/or depressive symptoms [33], or connectivity with frontal areas after F3/F4 tDCS in healthy adults [34]. Therefore, the secondary exploratory aim of this study was to record tDCS-related changes in EEG, and this way help illuminate the potential mechanism of action of tDCS, as well as the underlying pathology of neuropsychiatric symptoms of PCS. MS change and functional connectivity (FC) change between the frontal areas (F3/F4, i.e., at the stimulation site) were selected for analysis, and are the subject of this Paper.

## Methods

### Trial design

This was a randomised two-arm sham-controlled trial with 1:1 allocation ratio.

### Subjects

The study was registered in ISRCTN database (www.isrctn.com), under No.: ISRCTN10942585. The study was registered after the beginning of the enrolment (start of enrolment – 15/3/2022; registration date – 6/5/2022), as the registration took place after securing financing for the study. The study protocol, eligibility criteria, intervention procedures, and primary and secondary outcomes had been finalized before the start of the study. The study was approved by National Institute of Mental Health (NIMH) Ethics Committee on 16/11/2021, under the reference number 169/21. All participants signed an informed consent to participation in the study. The authors confirm that all ongoing and related trials for this intervention are registered. For participant inclusion, a PCS limit of >1 month, corresponding to a pre-existing paper applying tDCS (2), was chosen. Inclusion criteria were: age 18–75 years; fatigue or brain fog for > 1 month after detection of COVID-19 by a PCR test; Fatigue Impact Scale questionnaire score ≥ 40; presence of neuropsychiatric symptoms of PSC with Post-COVID-19 Symptoms Assessment Questionnaire A-PACS [2] total score ≥ 25; psychopharmacological medication on a stable dose for ≥ 4 weeks; competence to give informed consent. Exclusion criteria were: contraindications of tDCS; history of any other DSM-IV axis I diagnosis prior to COVID-19, except for depressive disorders, anxiety disorders, substance use disorders, and sleep disorders that may be present, but with at least 6 months of documented remission before entering the study; pregnancy or breastfeeding; severe and/or unstable internal or neurological disorders [23].

### Sample size

**Fig 1:**
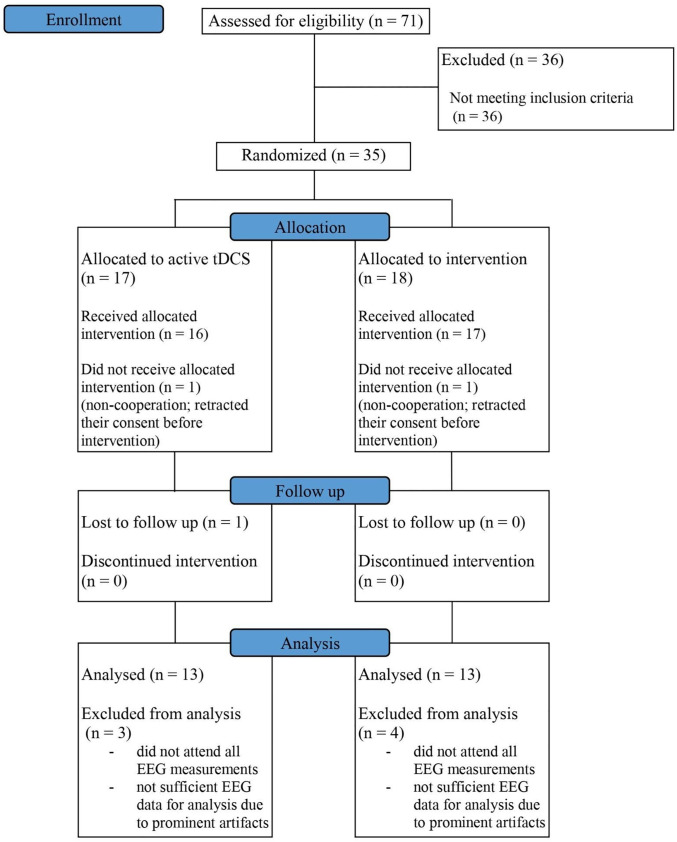
CONSORT diagram.

As at the time of designing the study, no other randomised control trials using tDCS in PCS were available (see below – tDCS application), it was decided to include n = 35 participants. The sample size was determined by institutional capacity. Two participants were screened and assigned to a particular group, but withdrew their consent prior to the tDCS intervention. Therefore, 33 participants were included, randomised into active (n = 16) and sham (n = 17) groups, and subsequently underwent the assigned intervention. Finally, 26 participants (n = 13 in active, n = 13 in sham group) were included in the EEG analyses; among them were 8 men and 18 women, mean age was 42 ± 10 years. Reasons for not performing EEG analysis in patients who underwent tDCS intervention included: not attending EEG measurement (n = 3) and not sufficient amount of EEG data remaining after rejection of artefacts and pre-processing (n = 4); for complete study diagram see Fig. 1. Only the final sample dataset of 26 patients was subsequently analysed, as the EEG analyses were exploratory, and not correlated to the clinical outcome.

### tDCS application

Participants received 20 sessions of tDCS, with duration of 30 min including 30s ramp-up and ramp-down phase at the beginning and at the end, current intensity of 2mA (current density 0.08mA/cm2), and electrodes montage over F3/F4 (anode-cathode, respectively). Sham-tDCS setup involved a 30s ramp-up phase at the beginning, followed by immediate ramp-down phase, and subsequent 29 min of rest. This stimulation protocol was chosen in accordance with guidelines and clinical trials with patients with similar symptoms: tDCS targeted at left DLPFC is effective in improving clinical status of patients with depressive symptoms [16], and fatigue [35], as well as cognitive functions in patients [36] and healthy adults [37].

TDCS was administered via HDCStim programmable stimulator (Newronica, Italy) and Mindcap (Newronica, Italy) electrode placement cap. This cap includes elliptically shaped sponge electrodes with size 25 cm². These were treated with a standard saline (0,9% NaCl). Stimulation was performed at home by the participants themselves, five times per week. The first application was performed at the clinic, in order to train the patients to perform it subsequently themselves. The Mindcap electrode placement cap is pre-made for patient-controlled home use. It includes built-in parts, which correspond to F3/F4 electrodes, therefore the patient only has to put the whole cap on their head. Furthermore, the correct placement was checked via video-call with a researcher. The stimulators retained the details of each stimulation (time, potential error, type of error), which were reviewed by a researcher after the completion of the protocol, or at the given moment in case of any malfunction. In case of potential adverse events, a contact for the researchers was provided.

### EEG acquisition

Standard 36-channel resting-state EEG was recorded before the start of intervention, after two weeks, and after the end of intervention (four weeks). EEG data were acquired by M&I BioSDA09 biosignal amplifier system with a 1kHz sampling rate, 24-bit sample resolution, analogue high pass filter at 0.015 Hz, anti-aliasing filter at 500 Hz. EEG electrodes were distributed by standard system 10−10, with ECG and EOG. Subjects were instructed to be in a relaxed state during recording. All recordings began with a 10-minute resting state with eyes closed followed by a 5-minute resting state with eyes open.

### Pre-processing

A commonly used sample of 5 min of recordings with eyes closed (minutes 1–6) was selected for all analyses. The 10-minute recording time was used in order to obtain enough EEG material in case of prominent artefacts; as this was eventually not the case, only minutes 1–6 (after deleting the beginning and end) were used. Pre-processing of EEG data was performed using the automatic pipeline TDBRAIN [38], which is implemented in Python. The pipeline was slightly modified for the specific characteristics of subsequent data analysis. The data were filtered using a band-pass FIR filter with cut-off frequencies of 1–40 Hz, and Hamming window. Before the automatic TDBRAIN pipeline identified artefact segments, Independent Component Analysis (ICA) method was applied, specifically fastICA algorithm from the MNE toolbox [39]. Using ICA, underlying biological artefacts were suppressed. TDBRAIN then identified segments where technical and residual biological artefacts were present. The signal was then segmented into 1-second-long sections (1000 samples). Only segments that contained no artefacts were retained for further analysis.

### Microstate analysis

MS analysis was performed in MATLAB programming environment using EEGLAB software [40] and EEGLAB plugin by T. Koenig, version 1.2 [41]. Frequency range: 1.0–40.0 Hz was analysed. Only global field power (GFP) peaks were selected for clustering via k-means clustering algorithm.

In this study, the commonly used microstates parameters were used to characterize the temporal dynamics of EEG signals. Concretely, the duration, occurrence, contribution, and Global Explain Variance (GEV) were used for microstate description. Duration refers to the average time a microstate persists before transitioning to another state, reflecting the stability of neural processes. Occurrence quantifies how frequently a microstate appears within a given time window, indicating the rate of state recurrence. Contribution (also called coverage) measures the proportion of total recording time occupied by a specific microstate, reflecting its dominance in overall brain dynamics. Finally, GEV assesses the proportion of total EEG variance explained by each microstate, providing a measure of its representational significance [42].

### Functional connectivity analysis

EEG FC analysis was performed according to Amplitude Envelope Correlation (AEC) method [43] and implemented in MNE tool library [39]. This method uses the statistical dependence between the envelopes of two EEG signals. Before calculating the signal envelope using the Hilbert transform, the signals were orthogonalised by subtracting the linearly regressed signal from the other. This takes into account the fact that a signal from one source in the brain may propagate to multiple electrodes. Thus, the FC calculation is performed twice (for each electrode), the resulting two values are averaged. Electrode pair F3 and F4 was chosen for FC calculation. The signal from entire available frequency range (1–40 Hz) was used. For each 1-second segment, the value of FC between electrodes F3 and F4 was calculated. Subsequently, FC values of all 1-second segments were averaged, thus one FC value was obtained for each recording.

### Statistical analysis

#### Microstates.

**Fig 2:**
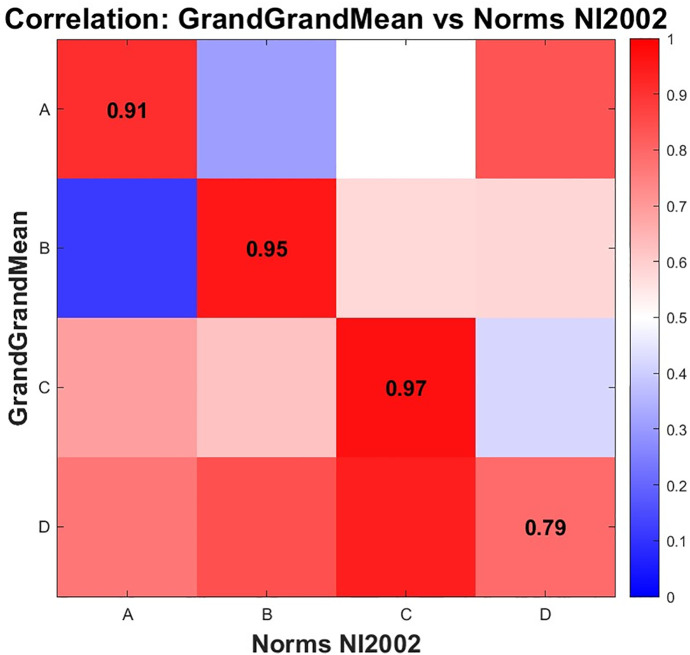
Microstate Pearson correlation of grand grand mean vs. normative template Norms NI2002, values higher than 0.7 which correspond to a strong correlation.

Average topographic maps were calculated from the whole ensemble, against which the topographic maps for each group were back-fitted. Backward fitting was performed based on global map dissimilarity (GMD). To ensure consistency of microstate labels (A, B, C, D) across groups and with normative templates (Norms NI2002), we applied a permutation-based template matching procedure, with all pairwise correlations between the spatial maps of grand grand mean dataset and normative microstate maps NI2002 [44]. Correlations were computed ignoring polarity. All possible permutations of the dataset maps relative to the template maps were generated and the permutation with the highest total sum of correlations was selected [44]. Correlation calculation was used to validate the fitting method used throughout the average topographic maps across the whole dataset. The values of Pearson correlation coefficients of the average topographic maps for each group and measurement reach values higher than 0.7, which corresponds to a strong correlation and confirms the appropriateness of using average topographic maps across the entire test set (see also Fig 2). Change after two weeks of treatment against the baseline measurement was assessed by subtracting microstate parameters values for baseline measurements from the parameter values after weeks of treatment. Subsequently, this was compared between the active vs. sham group. Similarly, change after the treatment (four weeks) against baseline was assessed and compared between active vs. sham group. Two hypotheses were posed for statistical testing: 1) *H0: There is no difference in change of MS parameters from baseline to measurement after two weeks between patients in active and sham group,* 2) *H0: There is no difference in change of MS parameters from baseline to measurement after four weeks (end-of-treatment) between patients in active and sham group.* Prior to statistical testing, the assumptions of normality and homogeneity of variances were assessed for all microstate variables using the Lilliefors and Levene’s tests, respectively. Because a substantial portion of the parameters violated the assumption of normal distribution (14 out of 48 evaluated comparisons across both time points), the non-parametric Mann-Whitney U test was used to evaluate differences in microstate parameter changes between the active and sham groups. To control for multiple testing, a Bonferroni correction was applied. Given the structure of microstate data, the correction was calculated for 12 temporal parameters (4 microstates × 3 parameters: duration, occurrence, coverage) and separately for 12 possible transitions, resulting in an adjusted significance threshold of α = 0.0042. Furthermore, effect sizes were calculated as $\text{r }=\;\frac{Z}{N}$ (where Z is the standardized test statistic and N is the total sample size) and 95% confidence intervals (CI) for the mean differences were computed.

#### Functional connectivity.

Prior to the statistical analysis of relative functional connectivity, the data were screened for outliers. Tukey’s method for outlier detection with a coefficient of 1.5 was applied and did not identify any outliers; this was further confirmed by visual inspection. The complete dataset obtained from 26 subjects was included in the subsequent analyses.

Two main hypotheses were statistically tested: *1) H0: There is no difference in FC change from baseline to measurement between active and sham groups, 2) H0: There is no difference in FC change from baseline between measurement after two weeks and after four weeks. Due to the mixed design, the third hypothesis was also tested: 3) H0: There is no interaction between group and time.* Statistical comparisons were performed using a two-factor mixed-design ANOVA, with time (measurement after 2 weeks vs. after 4 weeks) as the within-subject factor and groups (sham vs. active) as the between-subject factor. The normality of the data was assessed using the Shapiro–Wilk test, supported by visual inspection of Q–Q plots and density plots, revealing no statistically significant deviations from normality. No substantial deviations from homogeneity of variances were observed for the between-subject factor, as assessed by Levene’s test and visual inspection. Sphericity was not assessed for the within-subject factor, as it comprised only two levels. Given that all relevant assumptions were met, the two-factor mixed-design ANOVA was used to evaluate differences in relative functional connectivity.

### Outcomes

The primary outcome of this study was a clinical marker – change in the Fatigue Impact Scale at the end of treatment, analysed in the intention-to-treat population. The primary and further clinical and cognitive scales as secondary outcome are described in a separate article [23]. The EEG changes were defined as secondary exploratory outcomes.

### Interim analyses and stopping guidelines

Not applicable.

### Randomisation

The 1:1 allocation to the 2 test groups randomisation was realised according to Efron’s biased coin design [45]. A separate person, who was not part of the clinical or analysis team, and did not participate in subject recruitment, matched subject numbers to the particular test group, and subsequently set the tDCS machines.

### Blinding

Neither participants nor researchers in contact with participants knew, into which group each participant belonged; blinding was ensured by a third person, who set the tDCS machine (see above). The blinding success (i.e., evaluation of what the subjects thought was their assigned group) was not evaluated.

## Results

**Fig 3:**
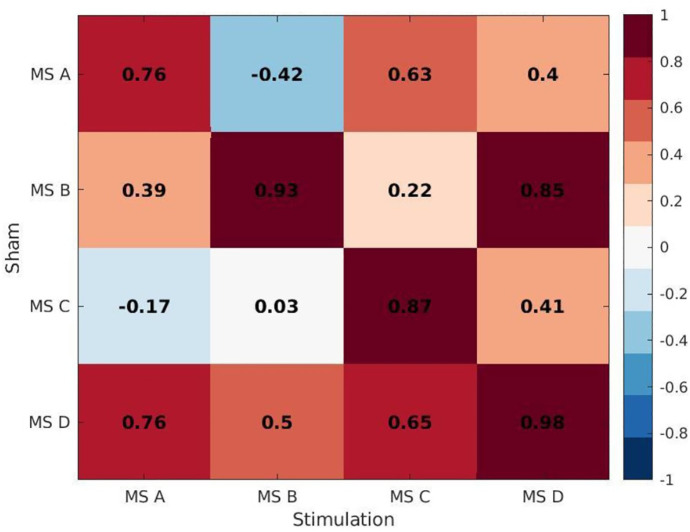
Correlation of topographic maps for particular microstates between active and sham groups at baseline (p-values are shown, active vs. grand mean correlated to sham vs. grand mean), documenting that the active and sham group did not differ at baseline.

**Fig 4:**
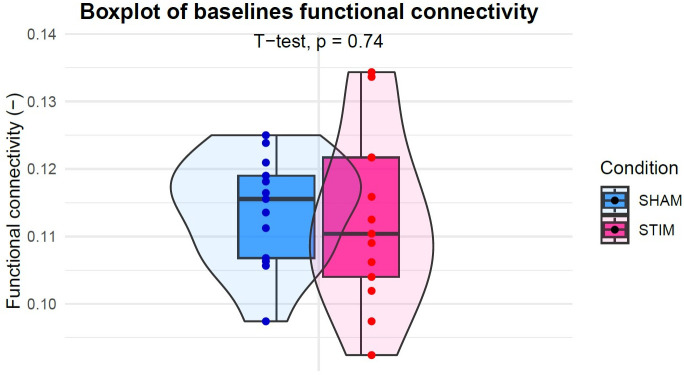
Baseline functional connectivity between the F3 and F4 electrode for active and sham group (difference is not statistically significant, p = 0,74).

Out of 71 screened participants, 35 met the inclusion criteria. Two of them withdrew before the start of intervention, therefore finally 33 patients (n = 16 in active, n = 17 in sham group) underwent the intervention and completed the study (i.e., completed all 20 sessions in the pre-specified time). All protocol completers received the full treatment (i.e., all 20 sessions in the allocated time of 4 weeks). There were no missed sessions. There were no major adherence issues or protocol deviations. Minor protocol deviations included postponing the application for 1–2 days (e.g., from a week-day to week-end), which had no effect on the total duration of the intervention. Device-recorded errors included pausing of the stimulation due to high impedance – a result of electrodes being insufficiently soaked in saline. Patients were trained prior to the intervention to resolve this issue by applying additional saline; each paused stimulation was resumed and finished normally. No other errors were noted. No serious adverse events were noted. All minor adverse events were expected (e.g., tingling or burning sensation), and did not influence the participants’ capability to continue with the stimulation. 26 participants were included in the EEG analyses (see Methods and Fig 1). There were no notable differences in demographic characteristics between the groups [23]. For basic demographic data see Table 1, for full details of patient characteristics, see Supplementary table S5 in S2 File. Small number of patients took medication that might potentially interfere with the effect of tDCS [46], specifically benzodiazepines (BZD; 6 patients, n = 4 in active group, n = 2 in sham group). However, the proportion of such patients did not differ between the groups, and patients were kept on stable medication over the course of the study. The particular groups also did not differ significantly (p = 0,76, p = 0,93, p = 0,87, p = 0,98 for MS A-D respectively; p = 0,74 for FC) at baseline measurement (see Fig 3 for MS and Fig 4 for FC).

**Table 1:** Demographic data (SD – standard deviation, F – female, A-PACS - Post-COVID-19 Symptoms Assessment Questionnaire).

Category	Active (n = 13)	Sham (n = 13)
**Age: mean (SD)**	44,30 (11,03)	40,30 (8,46)
**Sex: F**	9 (69%)	9 (69%)
**BMI: mean (SD)**	27,62 (6,58)	26,00 (7,89)
**Education (median)**	high school	high school
**postCOVID weeks: mean (SD)**	44,85 (30,24)	48,23 (21,43)
**The severity of COVID-19: mild/ moderate/ severe**	4/7/2	4/7/2
**History of psychiatric illness**	31%	46%
**Current use of psychiatric drugs**	38%	62%
**Antidepressants**	31%	62%
**Benzodiazepines/ Anticonvulsants**	8%	8%
**Baseline A-PACS: mean (SD)**	76,69 (19,06)	85,54 (24,98)

### Microstates

**Fig 5:**
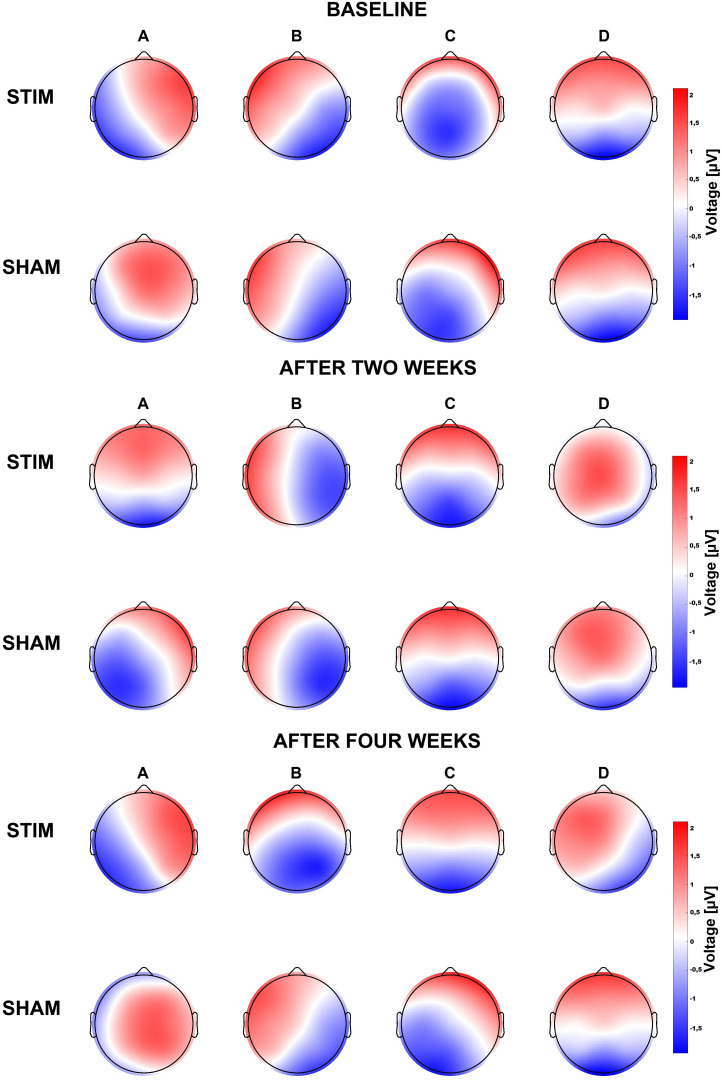
Microstate topographic maps for active and sham group at baseline, after two weeks, and after four weeks. These represent the electrical voltage distribution across the scalp for each microstate **(A – D)**. This visualisation reflects the absence of significant differences between the groups..

The following MS parameters were used for the evaluation: duration, occurrence, coverage, topographic maps, and transitions between MS. Mean global explained variance (GEV) at baseline, after two weeks and at end-of-treatment (after four weeks) was 69,82%, 67,51%, and 71,72% for the active group, and 69,87%, 68,98%, and 58,28% for the sham group. The mean MS duration is listed in Table 2. Microstate visualisation via mean topographic maps of both groups at each time point are shown in Fig 5. For full descriptive statistics of each MS parameter and MS topology correlations, see Supplementary material, Table S1 and Fig. S1-S9 in S1 File, respectively. No significant results emerged for either hypothesis tested. None of the group differences in microstate parameter changes (duration, occurrence, coverage, and transitions) reached statistical significance, even prior to the Bonferroni correction. The effect sizes (r) for all comparisons were small, further supporting the lack of significant differences between the active and sham groups. Therefore, the null hypotheses were not rejected. A summary of p-values is presented in Table 3, and complete details including exact p-values, effect sizes (r), and 95% confidence intervals for all microstate comparisons are comprehensively reported in Supplementary Table S2 and S3 in S1 File.

**Table 2:** Mean duration of each microstate (MS) for both active and sham group, at baseline, after two weeks, and after four weeks.

Duration (ms)		
	Baseline	
	Active	Sham
**MS A**	35.159	35.206
**MS B**	36.519	38.113
**MS C**	36.005	36.405
**MS D**	42.627	41.432
	**After two weeks**	
	**Active**	**Sham**
**MS A**	39.349	35.708
**MS B**	38.380	38.018
**MS C**	38.056	37.655
**MS D**	44.465	40.903
	**After four weeks**	
	**Active**	**Sham**
**MS A**	37.668	30.771
**MS B**	37.853	32.598
**MS C**	37.310	31.294
**MS D**	40.667	39.788

**Table 3:** Statistical analysis of difference in change in microstate properties after two and four weeks of treatment between active and sham groups. P-values are listed.

Change after two weeks					Change after four weeks				
Active vs. Sham					Active vs. Sham				
	**Coverage**	**Duration**	**Occurrence**	**GFP**		**Coverage**	**Duration**	**Occurrence**	**GFP**
**MS A**	0,182	0,682	0,538	0,918	**MS A**	0,166	0,412	0,473	0,798
**MS B**	0,573	1,000	0,798	0,878	**MS B**	0,644	0,878	0,959	0,798
**MS C**	0,644	0,918	0,959	0,959	**MS C**	0,644	0,837	0,918	0,720
**MS D**	0,918	0,758	0,644	0,959	**MS D**	0,505	0,758	0,259	0,918
Transitions					Transitions				
	**MS A**	**MS B**	**MS C**	**MS D**		**MS A**	**MS B**	**MS C**	**MS D**
**MS A**		0,238	0,878	0,608	**MS A**		0,282	0,383	1,000
**MS B**	0,282		0,573	0,918	**MS B**	0,305		0,798	0,720
**MS C**	0,918	0,305		0,758	**MS C**	0,356	0,798		0,238
**MS D**	0,442	1,000	0,383		**MS D**	0,878	0,720	0,383	

### Functional connectivity

**Fig 6:**
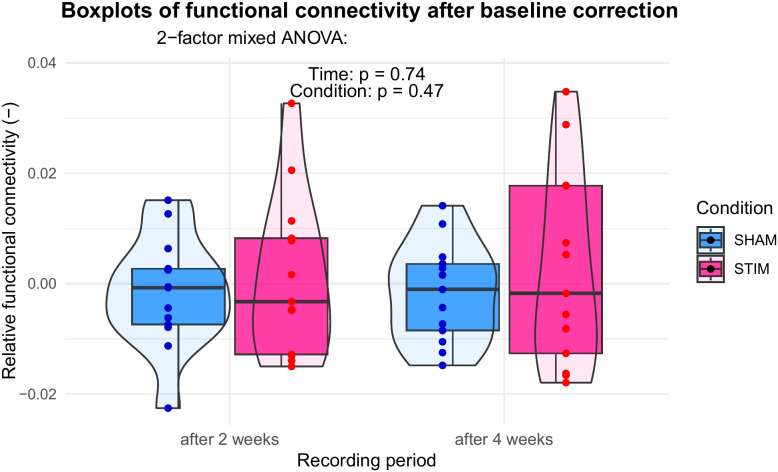
Boxplots of functional connectivity after baseline correction (i.e., the change from baseline to after-two-weeks and from baseline to after-four-weeks is shown).

Broadband frequency spectrum FC between electrodes F3 and F4 was evaluated at baseline (T0), after two weeks (T1) and at end-of-treatment (after four weeks, T2). Difference between T1 and T0 was: mean 0,001, SD 0,015 for active group, and mean −0,002, SD 0,01 for sham group. Difference between T2 and T0 was: mean 0,003, SD 0,018 for active group, and mean −0,002, SD 0,009 for sham group. The effects of group (active vs. sham), time (measurement after 2 weeks vs. after 4 weeks), and their interaction on changes in functional connectivity relative to baseline were examined using a two-factor mixed-design ANOVA. The analysis revealed no statistically significant main effect of group, F(1, 24) = 0.530, p = 0.473, η² = 0.0180, nor a significant main effect of time, F(1, 24) = 0.115, p = 0.737, η² = 0.0008. Additionally, the interaction between group and time was not statistically significant, F(1, 24) = 0.104, p = 0.750, η² = 0.0007, see Fig 6. The null hypotheses were not rejected (for differences in FC see Table 4, for full FC descriptive statistics, see Table S4 in S1 File).

**Table 4:** Descriptive statistics of functional connectivity change (SD – standard deviation, VAR – variance, MIN – minimal value, MAX – maximum value, 95% CI – 95% confidence interval).

Difference from baseline (T0) to after-two-weeks (T1) (difference T1-T0)							
	MEAN	MEDIAN	SD	VAR	MIN	MAX	95% CI
**ACTIVE**	0,001	−0,003	0,015	0,00021	−0,015	0,033	[−0,008, 0,010]
**SHAM**	−0,002	−0,001	0,010	0,00010	−0,023	0,015	[−0,008, 0,004]
**Difference from baseline (T0) to after-four-weeks (T2) (difference T2-T0)**							
	**MEAN**	**MEDIAN**	**SD**	**VAR**	**MIN**	**MAX**	**95% CI**
**ACTIVE**	0,003	−0,002	0,018	0,00032	−0,018	0,035	[−0,008, 0,013]
**SHAM**	−0,002	−0,001	0,009	0,00007	−0,015	0,014	[−0,007, 0,004]

Based on this analysis, it cannot be confirmed that stimulation (after two and four weeks) has an effect on the change in MS or FC between electrodes at stimulation sites. These results are consistent with clinical and cognitive status results, which also did not show a significant effect of stimulation.

## Discussion

In this RCT, we evaluated changes in certain EEG parameters after tDCS intervention in patients with psychiatric symptoms of PCS, specifically, change in duration, occurrence, contribution, and GEV of MS, and change in FC of areas directly related to neuromodulation – electrodes F3/F4. The effect of tDCS on the subjects’ cognitive and overall clinical status was also an outcome of this study and is described in a separate article by our research group [23]. We did not find any significant difference in FC or MS between the active tDCS and sham group, neither over the course of the intervention (week 2), nor at the end of the intervention (week 4). Interestingly, the sham group exhibited a descriptive reduction in GEV at T2 (~58%), compared with baseline and the active group (~70%). Although this difference was not statistically significant, it may reflect natural variability or nonspecific effects and warrants further investigation. The question arises, of whether these negative results reflect a “true negative”, i.e., that there is no effect of the stimulation, or whether the results are inconclusive due to limited power of the study. For primary outcome of the study, change in fatigue measured by Fatigue impact scale (FIS), a Bayesian factor was counted, with the result BF10 = 0,163 [23]. This means, that the observed data are 6,13 times more likely to occur under the *H0* hypothesis, which the Authors consider a true negative result. In the EEG results, no significant effects were observed, effect sizes were small (all effect sizes are below η² = 0.0180), which in connection with the clinical results, would imply a true negative interpretation. However, due to study’s limited precision, small effects cannot be definitively excluded. Several factors might contribute to the observed results.

One such factor is the choice of evaluated EEG parameters. MS were chosen for analysis, as a change of certain MS parameters was described in PCS patients [25]. However, as for MS analysis after a tDCS intervention, only a handful of studies, all of which included patients with other diagnoses or healthy volunteers, were realised. DLPFC and posterior parietal cortex tDCS ameliorated the pathological Microstate B in schizophrenia patients [30], orbitofrontal tDCS improved pathological transition between microstates A and C in obsessive-compulsive disorders patients [31], and certain MS changes were observed after a single session of DLPFC and dorsomedial prefrontal cortex tDCS in patients with depression [29]. Various measures of functional connectivity were affected by tDCS, such as a coherence index increase over left frontal regions after Fp1/F6 and TP1/AF3 (anode-cathode) tDCS in patients with anxiety and/or depressive symptoms [33], or fronto-parietal connectivity increase after F3/F4 tDCS [34], or increase between posterior cingulate cortex and right inferior parietal lobule after F3/P4 tDCS [47], however, the latter two included healthy population. For this study, the FC between the stimulation electrodes (F3/F4) was evaluated, as tDCS was found to change connectivity at the electrode sites [32,48]. Both MS and FC were chosen based on the information available during the creation of this study. However, as for the subsequent comparison between PCS patients and healthy adults, one study [49] found a disturbance in delta connectivity, as well as significant change in lower individual alpha frequency (IAF) and greater current source density (CSD) at the delta frequency band in bilateral frontal and central-temporal regions between Covid-19 patients within 2 months after hospital discharge and healthy controls. In a 10-month (period sufficient for the development of PCS) follow-up, the IAF was increased, but the CSD at delta frequency remained the same, suggesting a potential pathological finding in PCS. Other studies found a complexity reduction in frontal brain areas [50] and a general slowing of EEG in favour of delta [51] and theta [52] bands. In a recent RCT, tDCS was applied in PCS patients with concurrent EEG measurements (the first RCT to do so), with subsequent modulation of alpha band power [53]. In one case report [22], tDCS was applied in PCS patients, in 15 sessions over the dorsolateral prefrontal cortex (DLPFC; F3 anode, F4 cathode), with pre-/post-EEG recording. Changes in the power spectrum, i.e., delta-band power increase over F3 and decrease over F4, as well as alpha-, beta- and theta-band increase over both F3 and F4 were found. In our study, we did not analyse CSD, power spectral density, or complexity parameters, which might have potentially been affected by tDCS. In the FC analysis, the F3/F4 electrodes were chosen as regions of interest (ROI), corresponding to the stimulation electrodes position. Although tDCS might influence FC in these directly related ROIs [32,54,55], this study did not confirm this assumption. Choosing different ROIs, such as the fronto-parietal or temporo-parietal regions [56], might potentially yield observable results.

A brief mention should be dedicated to the concomitant medication of the patients during the intervention. Regular medication of a part of patients included drugs, which might influence EEG-measured brain activity, namely BZD, tricyclic antidepressants, or selective serotonin reuptake inhibitors [57]. Therefore, we may not completely rule out a contribution of their effect to the absence of significant EEG changes. Similarly, the medication of a small number of patients included BZD, which have been associated with a decrease of the tDCS effect in depression treatment [46]. However, although BZD use may influence the effect of tDCS in particular patients, as the total number of such cases was minimal, we regard the effect of BZD on group results as improbable. There was also a non-significant descriptive difference between the number of patients using BZD in active and sham group (6 patients, n = 4 in active group, n = 2 in sham group), which too is regarded as too small to produce any between-group difference. As the pre- and post-intervention EEG changes were analysed, while patients had to have stable doses of medication, effect of medication is unlikely.

Another factor might be electrode montage. TDCS was used as an intervention method for neuropsychiatric symptoms of PCS in two RCTs [58,59], none of which evaluated the change in EEG. However, the RCTs found an effect of tDCS on fatigue, as opposed to our study, with the electrode montage around the primary motor cortex – M1 area, roughly corresponding to the C3 electrode [58], or F3-Fp2 [59], respectively. Further two studies, where only an abstract is available, also found a reduction of fatigue after M1 tDCS [60,61]. Certain RCTs evaluated the efficacy of tDCS on other Covid-related symptoms and parameters, such as respiratory rehabilitation and days spent in intensive care unit [62], heart-rate-variability and autonomic functions [63], and hyposmia [64]. The electrode montage in these studies was: anode on left motor cortex with cathodes around [62]; anode on F3 and cathode on F4 – same as our study [63]; and anode on left PFC, cathode on contralateral shoulder [64], respectively. Currently running studies use the electrode montage: anode on the left motor cortex, cathode above the right orbit [65]; anode on F3 with cathodes around [66]; over the DLPFC [67,68]. Although our rationale for choosing the F3-F4 electrode montage was based on tDCS studies targeting similar symptoms in different diagnoses [16,19], other montages might also be effective.

One study [69] found a decrease of delta source activity bilaterally over frontal and parietal lobes and in the left temporal lobe in PCS patients, suggesting a suitable target area for tDCS intervention. F3-F4 montage did not yield any clinical results in our study and also in one case series [70], but resulted in fatigue alleviation in one case report [71]. Our choice of electrode montage was in accordance with this case report and also previous studies, in which the F3-F4 montage resulted in alleviation of depressive and anxiety symptoms in other diagnoses [72,73]. Therefore, we can assume that tDCS with a different electrode montage, with an alleviating effect on fatigue, might also result in changes in EEG.

Another noteworthy point is how tDCS can influence cognitive impairment and fatigue (and, in extension, the corresponding EEG changes) in PCS patients. It is suggested that fatigue and/or cognitive impairment in PCS patients are related to changes in GABAergic transmission [74–76]. TDCS is able to influence GABA levels and transmission: GABA levels were reduced in the left DLPFC with anodal tDCS (a-tDCS) at the F3 electrode, with further changes in striatal GABA levels [77], and in the left M1, with a-tDCS over M1 [78]. Another study also showed an a-tDCS-induced GABA decrease, which was connected with a decrease in resting-state functional coupling [79]. Animal studies show a GABA decrease under a-tDCS [80]. However, there is also a study that did not find any tDCS-induced change in GABA levels, albeit with a temporal electrode montage [81].

The question of what is the common underlying cause of EEG changes (greater delta band prevalence), GABA transmission disturbance, and accompanying clinical status (fatigue, worsening of cognitive functions) in PCS patients, remains unanswered. Potential pathological mechanisms include: a) direct damage of neural tissue by the virus, although presence of the virus during the post-acute period is not well researched [82]; b) hypoxia, which may induce slowing of EEG [51]; or c) autoimmune response, which may result into different forms of PCS [83].

Some similarities may be observed in other diagnoses with prevalent fatigue: in multiple sclerosis, the chronic fatigue was associated with frontoparietal dysfunction, similar to PCS [84], and was alleviated by tDCS [85]. In other studies, tDCS over the M1 area improved neuropathic pain in diabetes mellitus type 2 patients [86], and alleviated fibromyalgia symptoms [87].

It is also important to note, that this study was performed at a single geographical location, with a fairly homogenous population. Future studies with different populations might yield different results.

Although our study did not show that the tDCS intervention improved clinical symptoms or EEG parameters in PCS patients, certain implications for future studies may be drawn from studies with significant results. Firstly, patients who may benefit from tDCS intervention should be properly selected. EEG measurement is fast, cheap, and widely available. Moreover, standard qualitative visually inspected EEG is sufficient to identify general slowing and/or higher delta band prevalence, potentially filtering out patients in which electrophysiological changes are present. Next, the right target for tDCS should be selected – based on previous results and studies with other diagnoses, M1 area stimulation (corresponding to C3 electrode), or F3-C3 montage, or F3-Fp2 montage, subsequently affecting frontal and parietal regions [52,58,59], seem as suitable choices. However, given the heterogeneity regarding PCS diagnostic criteria, a great variety in duration of PCS and examination time points in previous studies, and the number of variable parameters during tDCS (electrode montage, current intensity, duration, number of sessions), further clinical research is needed. Protocols and results from studies with patients with fatigue and cognitive impairment based on other diagnoses, such as fibromyalgia [16,87], or multiple sclerosis [16,88] might serve as useful guidelines.

## Conclusion

In this study, we sought to improve clinical symptoms (fatigue and cognitive impairment) in PCS patients and examine the accompanying EEG changes (specifically FC and MS) as an exploratory endpoint. We did not find any significant change in clinical status, nor in EEG parameters. These results differ from those of other similar RCTs. Reasons for negative results of EEG parameters might include the choice of evaluated variables and tDCS parameters, however, due to exploratory nature of the study, powered primarily for clinical results, small effects cannot be definitely ruled out. Based on significant clinical results from other studies, future research with more suitable tDCS protocol and evaluated EEG variables might yield significant clinical outcomes and also help illuminate the underlying pathological mechanisms in PCD.

## Supporting information

S1 File Additional tables and figures. (DOCX)

S2 File Table S5 Full details of patient characteristics. (XLSX)

S3 File Study protocol. (PDF)

S4 File Study protocol Czech. (PDF)

S5 File CONSORT_2025_checklist. (DOCX)
