# Supplementary material for: Transcranial direct current stimulation in affecting neuropsychiatric symptoms of post-COVID syndrome: No change in microstates and functional connectivity
Source: PLoS One. 2026 Jun 26;21(6):e0351407. doi: 10.1371/journal.pone.0351407 (PMC13308831; doi:10.1371/journal.pone.0351407)

**Supplementary material**

TDCS intervention in neuropsychiatric symptoms of post-covid syndrome: Microstates and functional connectivity evaluation

**Table S1** Descriptive statistics of particular microstate properties

|  |  |  |  |  |  |  |  |  |  |  |
| --- | --- | --- | --- | --- | --- | --- | --- | --- | --- | --- |
| **BASELINE** | | | |  |  | **BASELINE** | | | |  |
| **ACTIVE** | | | |  |  | **SHAM** | | | |  |
| **Occurence (s^-1^)** | | | |  |  | **Occurence (s^-1^)** | | | |  |
|  | **Mean** | **Median** | **SD** |  |  |  | **Mean** | **Median** | **SD** |  |
| **MS 1** | 6,403 | 6,437 | 1,585 |  |  | **MS 1** | 6,355 | 6,150 | 1,522 |  |
| **MS 2** | 6,983 | 7,050 | 2,157 |  |  | **MS 2** | 7,160 | 7,308 | 1,633 |  |
| **MS 3** | 6,859 | 6,112 | 2,467 |  |  | **MS 3** | 6,780 | 6,284 | 2,183 |  |
| **MS 4** | 7,073 | 7,007 | 1,418 |  |  | **MS 4** | 6,626 | 6,331 | 1,322 |  |
|  |  |  |  |  |  |  |  |  |  |  |
| **Duration (ms)** | | | |  |  | **Duration (ms)** | | | |  |
|  | **Mean** | **Median** | **SD** |  |  |  | **Mean** | **Median** | **SD** |  |
| **MS 1** | 35,159 | 38,342 | 8,006 |  |  | **MS 1** | 35,206 | 34,392 | 6,088 |  |
| **MS 2** | 36,519 | 37,231 | 6,006 |  |  | **MS 2** | 38,113 | 39,301 | 6,006 |  |
| **MS 3** | 36,005 | 36,181 | 6,340 |  |  | **MS 3** | 36,405 | 36,181 | 5,869 |  |
| **MS 4** | 42,627 | 40,236 | 12,033 |  |  | **MS 4** | 41,432 | 40,236 | 12,033 |  |
|  |  |  |  |  |  |  |  |  |  |  |
| **Coverage (-)** | | | |  |  | **Coverage (-)** | | | |  |
|  | **Mean** | **Median** | **SD** |  |  |  | **Mean** | **Median** | **SD** |  |
| **MS 1** | 0,221 | 0,209 | 0,064 |  |  | **MS 1** | 0,220 | 0,230 | 0,049 |  |
| **MS 2** | 0,247 | 0,252 | 0,052 |  |  | **MS 2** | 0,267 | 0,262 | 0,044 |  |
| **MS 3** | 0,238 | 0,226 | 0,060 |  |  | **MS 3** | 0,242 | 0,226 | 0,065 |  |
| **MS 4** | 0,295 | 0,305 | 0,085 |  |  | **MS 4** | 0,272 | 0,260 | 0,080 |  |
|  |  |  |  |  |  |  |  |  |  |  |
| **GFP** | | | |  |  | **GFP** | | | |  |
|  | **Mean** | **Median** | **SD** |  |  |  | **Mean** | **Median** | **SD** |  |
| **MS 1** | 3,834 | 3,803 | 1,144 |  |  | **MS 1** | 3,798 | 3,922 | 0,761 |  |
| **MS 2** | 3,830 | 3,874 | 1,032 |  |  | **MS 2** | 3,870 | 3,970 | 0,715 |  |
| **MS 3** | 3,910 | 4,145 | 1,096 |  |  | **MS 3** | 3,904 | 4,066 | 0,692 |  |
| **MS 4** | 4,253 | 3,996 | 1,311 |  |  | **MS 4** | 4,241 | 4,232 | 0,843 |  |
|  |  |  |  |  |  |  |  |  |  |  |
|  | **Mean** | **Median** | **SD** |  |  |  | **Mean** | **Median** | **SD** |  |
| **GEV** | 69,82% | 70,98% | 8,64% |  |  | **GEV** | 69,87% | 71,62% | 7,74% |  |
|  |  |  |  |  |  |  |  |  |  |  |
| **Transitions, Mean Values** | | | | |  | **Transitions, Mean Values** | | | | |
|  | **MS 1** | **MS 2** | **MS 3** | **MS 4** |  |  | **MS 1** | **MS 2** | **MS 3** | **MS 4** |
| **MS 1** |  | 0,075 | 0,075 | 0,086 |  | **MS 1** |  | 0,082 | 0,075 | 0,079 |
| **MS 2** | 0,077 |  | 0,084 | 0,092 |  | **MS 2** | 0,082 |  | 0,092 | 0,091 |
| **MS 3** | 0,073 | 0,086 |  | 0,088 |  | **MS 3** | 0,076 | 0,093 |  | 0,081 |
| **MS 4** | 0,085 | 0,092 | 0,088 |  |  | **MS 4** | 0,078 | 0,090 | 0,082 |  |
|  |  |  |  |  |  |  |  |  |  |  |
| Transitions | | | | |  | Transitions | | | | |
|  | **MS 1** | **MS 2** | **MS 3** | **MS 4** |  |  | **MS 1** | **MS 2** | **MS 3** | **MS 4** |
| **MS 1** | MS1->MS1 | MS2->MS1 | MS3->MS1 | MS4->MS1 |  | **MS 1** | MS1->MS1 | MS2->MS1 | MS3->MS1 | MS4->MS1 |
| **MS 2** | MS1->MS2 | MS2->MS2 | MS3->MS2 | MS4->MS2 |  | **MS 2** | MS1->MS2 | MS2->MS2 | MS3->MS2 | MS4->MS2 |
| **MS 3** | MS1->MS3 | MS2->MS3 | MS3->MS3 | MS4->MS3 |  | **MS 3** | MS1->MS3 | MS2->MS3 | MS3->MS3 | MS4->MS3 |
| **MS 4** | MS1->MS4 | MS2->MS4 | MS3->MS4 | MS4->MS4 |  | **MS 4** | MS1->MS4 | MS2->MS4 | MS3->MS4 | MS4->MS4 |

|  |  |  |  |  |  |  |  |  |  |
| --- | --- | --- | --- | --- | --- | --- | --- | --- | --- |
| **AFTER TWO WEEKS** | | | |  | **AFTER TWO WEEKS** | | | |  |
| **ACTIVE** | | | |  | **SHAM** | | | |  |
| **Occurence (s^-1^)** | | | |  | **Occurence (s^-1^)** | | | |  |
|  | **Mean** | **Median** | **SD** |  |  | **Mean** | **Median** | **SD** |  |
| **MS 1** | 6,573 | 6,257 | 1,765 |  | **MS 1** | 6,141 | 6,012 | 1,253 |  |
| **MS 2** | 6,131 | 6,559 | 1,447 |  | **MS 2** | 6,992 | 7,031 | 1,662 |  |
| **MS 3** | 6,093 | 5,921 | 1,365 |  | **MS 3** | 6,919 | 6,235 | 1,919 |  |
| **MS 4** | 6,262 | 6,358 | 1,365 |  | **MS 4** | 6,391 | 6,407 | 0,550 |  |
|  |  |  |  |  |  |  |  |  |  |
| **Duration (ms)** | | | |  | **Duration (ms)** | | | |  |
|  | **Mean** | **Median** | **SD** |  |  | **Mean** | **Median** | **SD** |  |
| **MS 1** | 39,349 | 38,619 | 5,465 |  | **MS 1** | 35,708 | 36,353 | 5,895 |  |
| **MS 2** | 38,380 | 38,050 | 6,040 |  | **MS 2** | 38,018 | 37,690 | 4,533 |  |
| **MS 3** | 38,056 | 38,006 | 5,403 |  | **MS 3** | 37,655 | 37,075 | 4,313 |  |
| **MS 4** | 44,465 | 44,470 | 13,118 |  | **MS 4** | 40,903 | 39,694 | 11,208 |  |
|  |  |  |  |  |  |  |  |  |  |
| **Coverage (-)** | | | |  | **Coverage (-)** | | | |  |
|  | **Mean** | **Median** | **SD** |  |  | **Mean** | **Median** | **SD** |  |
| **MS 1** | 0,257 | 0,238 | 0,071 |  | **MS 1** | 0,218 | 0,217 | 0,053 |  |
| **MS 2** | 0,233 | 0,251 | 0,053 |  | **MS 2** | 0,263 | 0,262 | 0,059 |  |
| **MS 3** | 0,230 | 0,226 | 0,067 |  | **MS 3** | 0,257 | 0,252 | 0,061 |  |
| **MS 4** | 0,280 | 0,294 | 0,091 |  | **MS 4** | 0,262 | 0,265 | 0,076 |  |
|  |  |  |  |  |  |  |  |  |  |
| **GFP** | | | |  | **GFP** | | | |  |
|  | **Mean** | **Median** | **SD** |  |  | **Mean** | **Median** | **SD** |  |
| **MS 1** | 3,788 | 3,650 | 1,745 |  | **MS 1** | 3,865 | 3,563 | 1,186 |  |
| **MS 2** | 3,772 | 3,597 | 1,646 |  | **MS 2** | 3,931 | 3,561 | 1,130 |  |
| **MS 3** | 3,800 | 3,654 | 1,661 |  | **MS 3** | 3,986 | 3,493 | 1,200 |  |
| **MS 4** | 4,233 | 4,146 | 1,926 |  | **MS 4** | 4,320 | 3,927 | 1,418 |  |
|  |  |  |  |  |  |  |  |  |  |
|  | **Mean** | **Median** | **SD** |  |  | **Mean** | **Median** | **SD** |  |
| **GEV** | 67,51% | 71,82% | 20,01% |  | **GEV** | 68,98% | 72,10% | 6,49% |  |
|  |  |  |  |  |  |  |  |  |  |
| **Transitions, Mean Values** | | | | | **Transitions, Mean Values** | | | | |
|  | **MS 1** | **MS 2** | **MS 3** | **MS 4** |  | **MS 1** | **MS 2** | **MS 3** | **MS 4** |
| **MS 1** |  | 0,083 | 0,088 | 0,089 | **MS 1** |  | 0,080 | 0,080 | 0,073 |
| **MS 2** | 0,082 |  | 0,076 | 0,085 | **MS 2** | 0,080 |  | 0,093 | 0,089 |
| **MS 3** | 0,088 | 0,074 |  | 0,080 | **MS 3** | 0,079 | 0,095 |  | 0,084 |
| **MS 4** | 0,091 | 0,086 | 0,077 |  | **MS 4** | 0,074 | 0,087 | 0,085 |  |
|  |  |  |  |  |  |  |  |  |  |
|  |  |  |  |  |  |  |  |  |  |
| Transitions | | | | | Transitions | | | | |
|  | **MS 1** | **MS 2** | **MS 3** | **MS 4** |  | **MS 1** | **MS 2** | **MS 3** | **MS 4** |
| **MS 1** | MS1->MS1 | MS2->MS1 | MS3->MS1 | MS4->MS1 | **MS 1** | MS1->MS1 | MS2->MS1 | MS3->MS1 | MS4->MS1 |
| **MS 2** | MS1->MS2 | MS2->MS2 | MS3->MS2 | MS4->MS2 | **MS 2** | MS1->MS2 | MS2->MS2 | MS3->MS2 | MS4->MS2 |
| **MS 3** | MS1->MS3 | MS2->MS3 | MS3->MS3 | MS4->MS3 | **MS 3** | MS1->MS3 | MS2->MS3 | MS3->MS3 | MS4->MS3 |
| **MS 4** | MS1->MS4 | MS2->MS4 | MS3->MS4 | MS4->MS4 | **MS 4** | MS1->MS4 | MS2->MS4 | MS3->MS4 | MS4->MS4 |

|  |  |  |  |  |  |  |  |  |  |  |
| --- | --- | --- | --- | --- | --- | --- | --- | --- | --- | --- |
| **AFTER FOUR WEEKS** | | | |  |  | **AFTER FOUR WEEKS** | | | |  |
| **ACTIVE** | | | |  |  | **SHAM** | | | |  |
| **Occurence (s^-1^)** | | | |  |  | **Occurence (s^-1^)** | | | |  |
|  | **Mean** | **Median** | **SD** |  |  |  | **Mean** | **Median** | **SD** |  |
| **MS 1** | 6,402 | 6,281 | 1,246 |  |  | **MS 1** | 5,334 | 5,795 | 1,766 |  |
| **MS 2** | 6,911 | 6,407 | 1,411 |  |  | **MS 2** | 5,864 | 6,908 | 1,545 |  |
| **MS 3** | 6,754 | 6,252 | 1,551 |  |  | **MS 3** | 5,535 | 6,681 | 1,229 |  |
| **MS 4** | 6,373 | 6,295 | 0,967 |  |  | **MS 4** | 5,724 | 6,464 | 1,234 |  |
|  |  |  |  |  |  |  |  |  |  |  |
| **Duration (ms)** | | | |  |  | **Duration (ms)** | | | |  |
|  | **Mean** | **Median** | **SD** |  |  |  | **Mean** | **Median** | **SD** |  |
| **MS 1** | 37,668 | 36,020 | 7,664 |  |  | **MS 1** | 30,771 | 33,188 | 4,465 |  |
| **MS 2** | 37,853 | 38,648 | 5,636 |  |  | **MS 2** | 32,598 | 38,592 | 4,977 |  |
| **MS 3** | 37,310 | 36,968 | 4,906 |  |  | **MS 3** | 31,294 | 37,604 | 6,537 |  |
| **MS 4** | 40,667 | 39,968 | 11,871 |  |  | **MS 4** | 39,788 | 39,849 | 15,443 |  |
|  |  |  |  |  |  |  |  |  |  |  |
| **Coverage (-)** | | | |  |  | **Coverage (-)** | | | |  |
|  | **Mean** | **Median** | **SD** |  |  |  | **Mean** | **Median** | **SD** |  |
| **MS 1** | 0,240 | 0,225 | 0,062 |  |  | **MS 1** | 0,187 | 0,222 | 0,056 |  |
| **MS 2** | 0,257 | 0,273 | 0,045 |  |  | **MS 2** | 0,214 | 0,258 | 0,066 |  |
| **MS 3** | 0,247 | 0,227 | 0,049 |  |  | **MS 3** | 0,197 | 0,237 | 0,059 |  |
| **MS 4** | 0,256 | 0,242 | 0,089 |  |  | **MS 4** | 0,263 | 0,258 | 0,096 |  |
|  |  |  |  |  |  |  |  |  |  |  |
| **GFP** | | | |  |  | **GFP** | | | |  |
|  | **Mean** | **Median** | **SD** |  |  |  | **Mean** | **Median** | **SD** |  |
| **MS 1** | 3,741 | 3,699 | 1,175 |  |  | **MS 1** | 3,508 | 3,766 | 1,260 |  |
| **MS 2** | 3,735 | 3,817 | 1,175 |  |  | **MS 2** | 3,515 | 3,978 | 1,184 |  |
| **MS 3** | 3,778 | 3,802 | 1,175 |  |  | **MS 3** | 3,609 | 4,072 | 1,269 |  |
| **MS 4** | 4,104 | 4,138 | 1,404 |  |  | **MS 4** | 3,917 | 4,302 | 1,492 |  |
|  |  |  |  |  |  |  |  |  |  |  |
|  | **Mean** | **Median** | **SD** |  |  |  | **Mean** | **Median** | **SD** |  |
| **GEV** | 71,72% | 72,15% | 5,42% |  |  | **GEV** | 58,28% | 66,99% | 6,47% |  |
|  |  |  |  |  |  |  |  |  |  |  |
|  |  |  |  |  |  |  |  |  |  |  |
| **Transitions, Mean Values** | | | | |  | **Transitions, Mean Values** | | | | |
|  | **MS 1** | **MS 2** | **MS 3** | **MS 4** |  |  | **MS 1** | **MS 2** | **MS 3** | **MS 4** |
| **MS 1** |  | 0,085 | 0,082 | 0,078 |  | **MS 1** |  | 0,067 | 0,062 | 0,074 |
| **MS 2** | 0,083 |  | 0,089 | 0,087 |  | **MS 2** | 0,067 |  | 0,074 | 0,079 |
| **MS 3** | 0,082 | 0,089 |  | 0,080 |  | **MS 3** | 0,061 | 0,074 |  | 0,075 |
| **MS 4** | 0,079 | 0,084 | 0,081 |  |  | **MS 4** | 0,074 | 0,080 | 0,074 |  |
|  |  |  |  |  |  |  |  |  |  |  |
|  |  |  |  |  |  |  |  |  |  |  |
| Transitions | | | | |  | Transitions | | | | |
|  | **MS 1** | **MS 2** | **MS 3** | **MS 4** |  |  | **MS 1** | **MS 2** | **MS 3** | **MS 4** |
| **MS 1** | MS1->MS1 | MS2->MS1 | MS3->MS1 | MS4->MS1 | | **MS 1** | MS1->MS1 | MS2->MS1 | MS3->MS1 | MS4->MS1 |
| **MS 2** | MS1->MS2 | MS2->MS2 | MS3->MS2 | MS4->MS2 | | **MS 2** | MS1->MS2 | MS2->MS2 | MS3->MS2 | MS4->MS2 |
| **MS 3** | MS1->MS3 | MS2->MS3 | MS3->MS3 | MS4->MS3 | | **MS 3** | MS1->MS3 | MS2->MS3 | MS3->MS3 | MS4->MS3 |
| **MS 4** | MS1->MS4 | MS2->MS4 | MS3->MS4 | MS4->MS4 |  | **MS 4** | MS1->MS4 | MS2->MS4 | MS3->MS4 | MS4->MS4 |

**Table S2** Statistical analysis of Microstates – change after 2 weeks

|  | **Change after two weeks** | | | | |
| --- | --- | --- | --- | --- | --- |
| **Metric** | **Microstate** | **p_value** | **EffectSize_r** | **CI_Lower** | **CI_Upper** |
| Contribution | MS A | 0,182 | 0,261 | -0,026 | 0,101 |
| Contribution | MS B | 0,573 | 0,111 | -0,063 | 0,043 |
| Contribution | MS C | 0,644 | 0,091 | -0,080 | 0,034 |
| Contribution | MS D | 0,918 | 0,020 | -0,101 | 0,093 |
| Duration | MS A | 0,682 | 0,080 | -0,004 | 0,011 |
| Duration | MS B | 1,000 | 0,000 | -0,004 | 0,008 |
| Duration | MS C | 0,918 | 0,020 | -0,006 | 0,008 |
| Duration | MS D | 0,758 | 0,060 | -0,013 | 0,018 |
| Occurrence | MS A | 0,538 | 0,121 | -1,508 | 2,276 |
| Occurrence | MS B | 0,798 | 0,050 | -2,764 | 1,397 |
| Occurrence | MS C | 0,959 | 0,010 | -3,085 | 1,277 |
| Occurrence | MS D | 0,644 | 0,091 | -2,206 | 1,055 |
| MeanGFP | MS A | 0,918 | 0,020 | -1,377 | 1,151 |
| MeanGFP | MS B | 0,878 | 0,030 | -1,313 | 1,075 |
| MeanGFP | MS C | 0,959 | 0,010 | -1,426 | 1,042 |
| MeanGFP | MS D | 0,959 | 0,010 | -1,576 | 1,381 |
| Transition | MS A->B | 0,282 | 0,211 | -0,008 | 0,023 |
| Transition | MS A->C | 0,918 | 0,020 | -0,025 | 0,048 |
| Transition | MS A->D | 0,442 | 0,151 | -0,014 | 0,031 |
| Transition | MS B->A | 0,238 | 0,231 | -0,007 | 0,027 |
| Transition | MS B->C | 0,305 | 0,201 | -0,041 | 0,013 |
| Transition | MS B->D | 1,000 | 0,000 | -0,027 | 0,023 |
| Transition | MS C->A | 0,878 | 0,030 | -0,028 | 0,044 |
| Transition | MS C->B | 0,573 | 0,111 | -0,036 | 0,018 |
| Transition | MS C->D | 0,383 | 0,171 | -0,038 | 0,010 |
| Transition | MS D->A | 0,608 | 0,101 | -0,013 | 0,031 |
| Transition | MS D->B | 0,918 | 0,020 | -0,030 | 0,021 |
| Transition | MS D->C | 0,758 | 0,060 | -0,037 | 0,013 |

**Table S3** Statistical analysis of Microstates – change after 4 weeks

|  | **Change after four weeks** | | | | |
| --- | --- | --- | --- | --- | --- |
| **Metric** | **Microstate** | **p_value** | **EffectSize_r** | **CI_Lower** | **CI_Upper** |
| Contribution | MS A | 0,166 | 0,272 | -0,029 | 0,084 |
| Contribution | MS B | 0,644 | 0,091 | -0,052 | 0,059 |
| Contribution | MS C | 0,644 | 0,091 | -0,082 | 0,056 |
| Contribution | MS D | 0,505 | 0,131 | -0,129 | 0,093 |
| Duration | MS A | 0,412 | 0,161 | -0,003 | 0,011 |
| Duration | MS B | 0,878 | 0,030 | -0,005 | 0,009 |
| Duration | MS C | 0,837 | 0,040 | -0,006 | 0,008 |
| Duration | MS D | 0,758 | 0,060 | -0,016 | 0,018 |
| Occurrence | MS A | 0,473 | 0,141 | -1,928 | 2,110 |
| Occurrence | MS B | 0,959 | 0,010 | -2,270 | 1,692 |
| Occurrence | MS C | 0,918 | 0,020 | -2,993 | 1,838 |
| Occurrence | MS D | 0,259 | 0,221 | -2,204 | 0,795 |
| MeanGFP | MS A | 0,798 | 0,050 | -1,376 | 1,025 |
| MeanGFP | MS B | 0,798 | 0,050 | -1,300 | 1,029 |
| MeanGFP | MS C | 0,720 | 0,070 | -1,451 | 0,999 |
| MeanGFP | MS D | 0,918 | 0,020 | -1,623 | 1,245 |
| Transition | MS A->B | 0,305 | 0,201 | -0,007 | 0,029 |
| Transition | MS A->C | 0,356 | 0,181 | -0,014 | 0,032 |
| Transition | MS A->D | 0,878 | 0,030 | -0,028 | 0,026 |
| Transition | MS B->A | 0,282 | 0,211 | -0,008 | 0,029 |
| Transition | MS B->C | 0,798 | 0,050 | -0,041 | 0,031 |
| Transition | MS B->D | 0,720 | 0,070 | -0,026 | 0,024 |
| Transition | MS C->A | 0,383 | 0,171 | -0,015 | 0,029 |
| Transition | MS C->B | 0,798 | 0,050 | -0,040 | 0,031 |
| Transition | MS C->D | 0,383 | 0,171 | -0,033 | 0,009 |
| Transition | MS D->A | 1,000 | 0,000 | -0,026 | 0,028 |
| Transition | MS D->B | 0,720 | 0,070 | -0,027 | 0,024 |
| Transition | MS D->C | 0,238 | 0,231 | -0,034 | 0,007 |

**Table S4** Descriptive statistics of functional connectivity (SD - standard deviation, VAR - variance, MIN - minimal value, MAX - maximum value)

| FUNCTIONAL CONNECTIVITY AT BASELINE | | | | | | |
| --- | --- | --- | --- | --- | --- | --- |
| T0 | **MEAN** | **MEDIAN** | **SD** | **VAR** | **MIN** | **MAX** |
| ACTIVE | 0,112 | 0,110 | 0,013 | 0,00016 | 0,092 | 0,134 |
| SHAM | 0,113 | 0,116 | 0,008 | 0,00006 | 0,097 | 0,125 |
| FUNCTIONAL CONNECTIVITY AFTER TWO WEEKS | | | | | | |
| T1 | **MEAN** | **MEDIAN** | **SD** | **VAR** | **MIN** | **MAX** |
| ACTIVE | 0,114 | 0,117 | 0,009 | 0,00008 | 0,093 | 0,125 |
| SHAM | 0,112 | 0,115 | 0,011 | 0,00011 | 0,094 | 0,129 |
| FUNCTIONAL CONNECTIVITY AFTER FOUR WEEKS | | | | | | |
| T2 | **MEAN** | **MEDIAN** | **SD** | **VAR** | **MIN** | **MAX** |
| ACTIVE | 0,115 | 0,111 | 0,011 | 0,00012 | 0,099 | 0,133 |
| SHAM | 0,112 | 0,113 | 0,010 | 0,00010 | 0,097 | 0,130 |

**Figure S1** Correlation of microstate topology between active group and grand mean according to global map dissimilarity at baseline


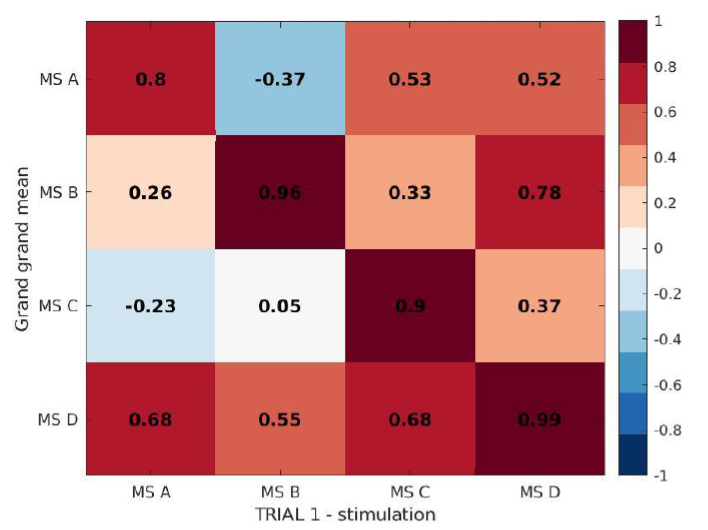


**Figure S2** Correlation of microstate topology between sham group and grand mean according to global map dissimilarity at baseline


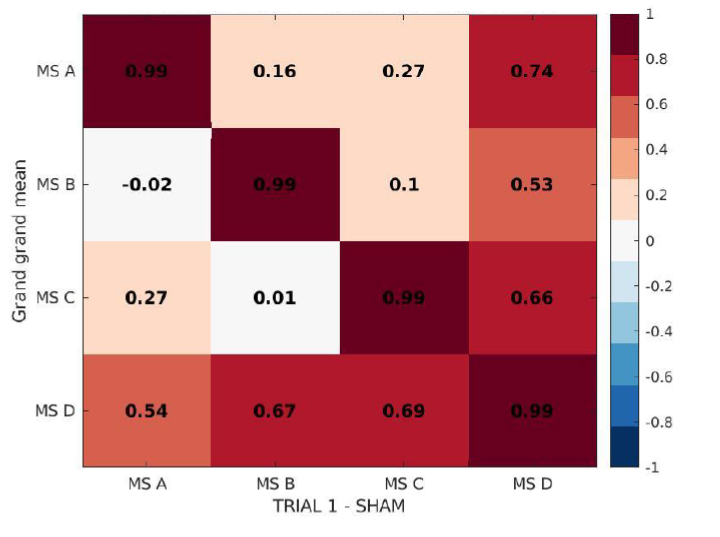


**Figure S3** Correlation of microstate topology at baseline (active vs. grand mean correlated to sham vs. grand mean)


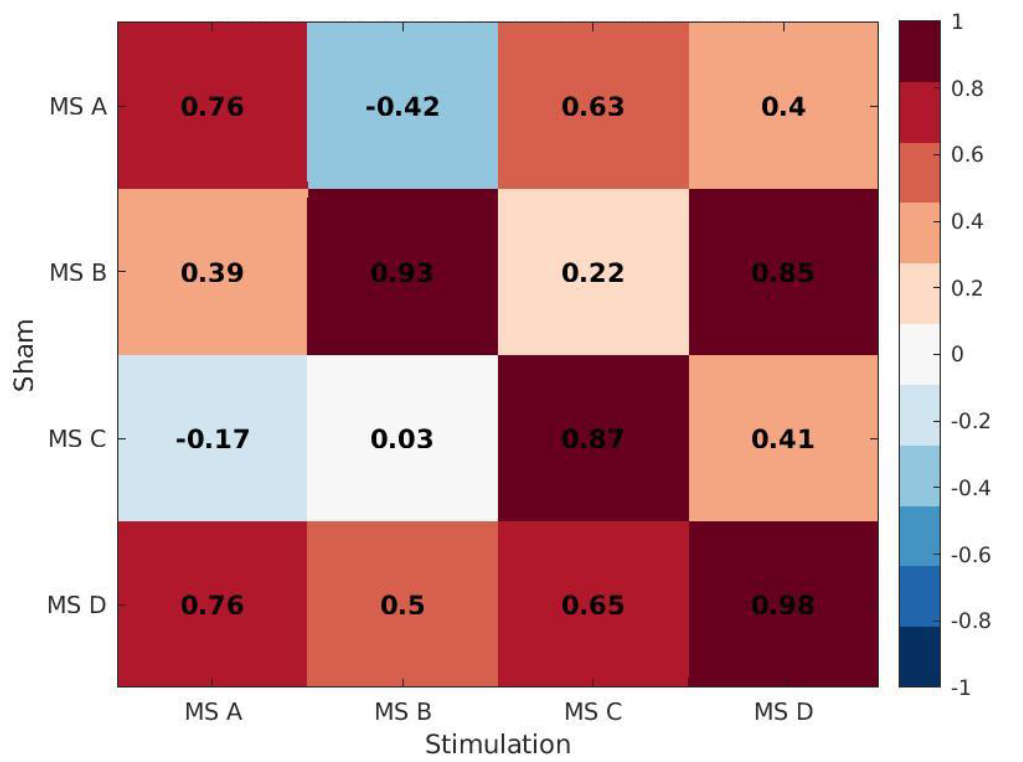


**Figure S4** Correlation of microstate topology between active group and grand mean according to global map dissimilarity after two weeks of treatment


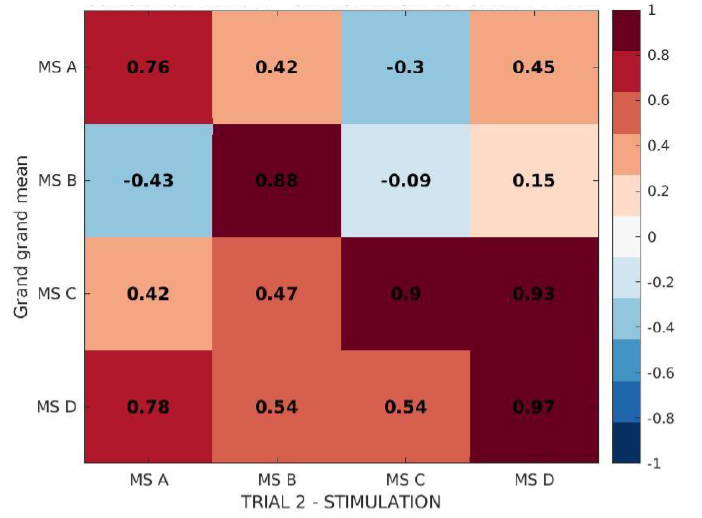


**Figure S5** Correlation of microstate topology between sham group and grand mean according to global map dissimilarity after two weeks of treatment


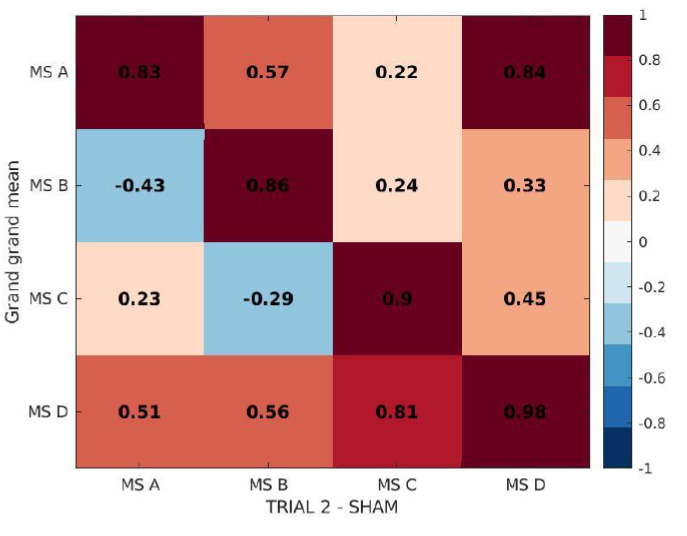


**Figure S6** Correlation of microstate topology after two weeks of treatment (active vs. grand mean correlated to sham vs. grand mean)


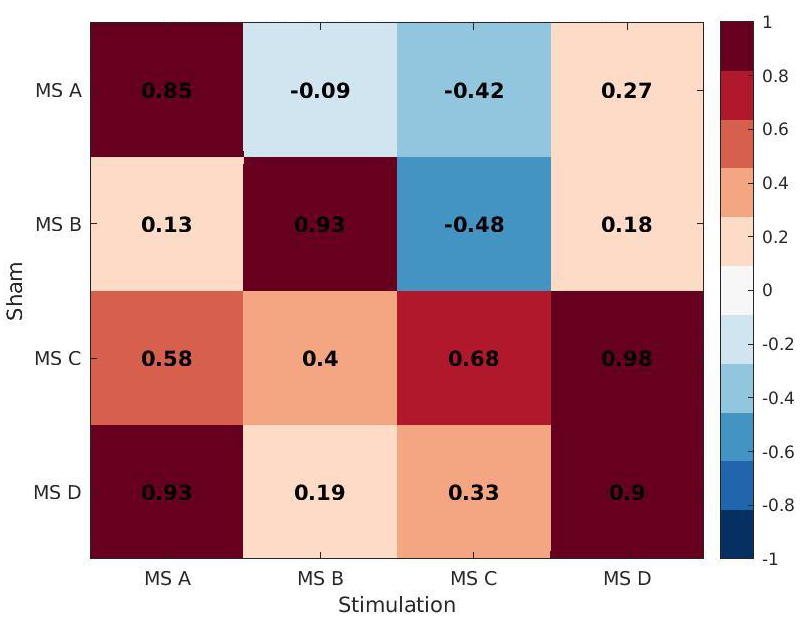


**Figure S7** Correlation of microstate topology between active group and grand mean according to global map dissimilarity after four weeks of treatment


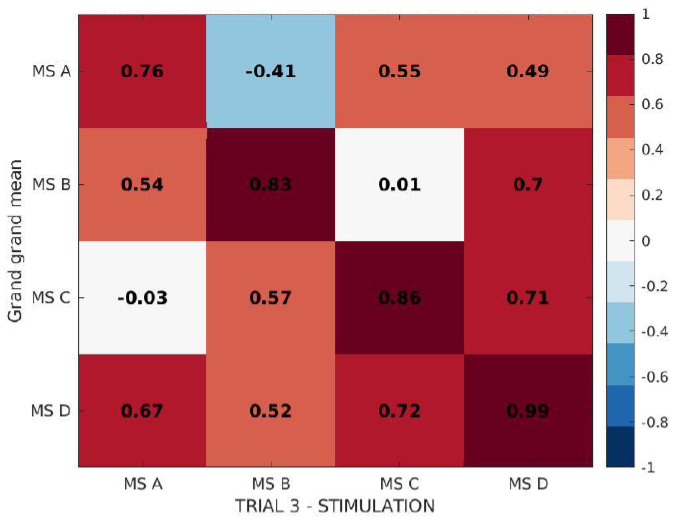


**Figure S8** Correlation of microstate topology between sham group and grand mean according to global map dissimilarity after four weeks of treatment


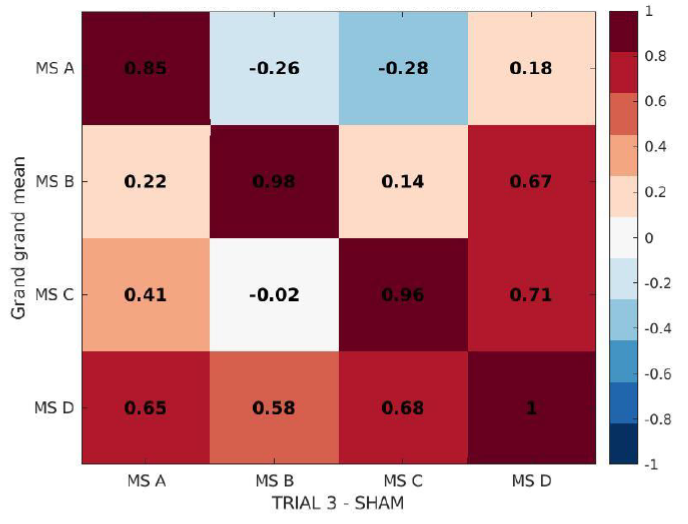


**Figure S9** Correlation of microstate topology after four weeks of treatment (active vs. grand mean correlated to sham vs. grand mean)


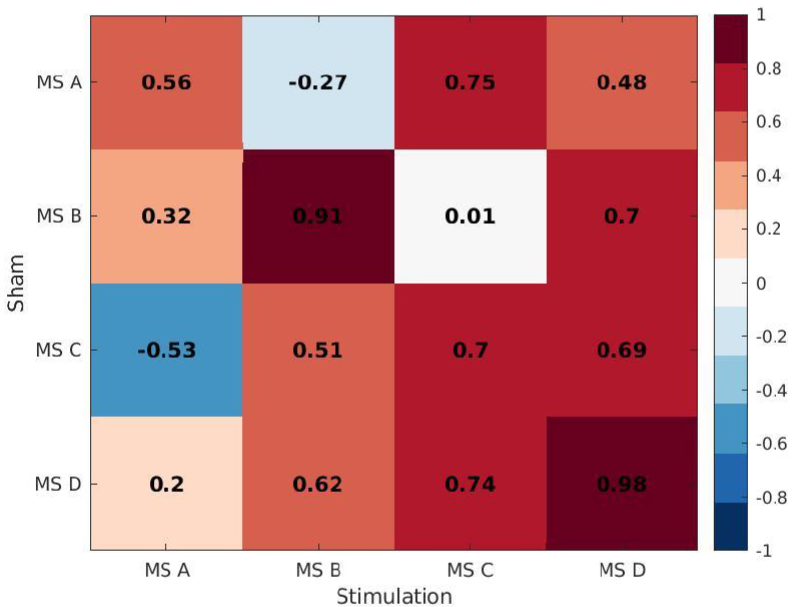

Supplement: S1 File — (DOCX) [file pone.0351407.s001.docx]
