# Supplementary material for: Transcranial direct current stimulation in affecting neuropsychiatric symptoms of post-COVID syndrome: No change in microstates and functional connectivity
Source: PLoS One. 2026 Jun 26;21(6):e0351407. doi: 10.1371/journal.pone.0351407 (PMC13308831; doi:10.1371/journal.pone.0351407)
Supplement: S3 File — (PDF) [file pone.0351407.s003.pdf]

PROJECT PROPOSAL

Name of the Project: **Transcranial direct current stimulation (tDCS) as a therapeutic intervention for post-acute sequelae of SARS-CoV-2 (PASC)**

Název projektu: **Transkraniální stimulace stejnosměrným proudem (tDCS) jako léčebná intervence pro zotavení z post-akutních následků SARS-CoV-2 (PASC)**

NUDZ Principal Investigator: Monika Klírová, M.D., Ph.D.

Aim of the study: The main aim of this project is to evaluate the clinical efficacy and safety of transcranial direct current stimulation (tDCS) of the dorsolateral prefrontal cortex (DLPFC) in the treatment of neuropsychiatric symptoms of the post-acute sequelae of SARS-CoV-2 infection (PASC). The primary objective of the double-blind, randomized, placebo-controlled, parallel study with four-week follow-up is to compare the efficacy (response to the treatment, effect duration) and safety of 4-week active DLPFC-tDCS (20 tDCS applications with anode placement at F3; 2 mA, 30 min/tDCS session) to placebo-tDCS in the treatment of neuropsychiatric symptoms of PASC. The primary study outcome is a change in validated Czech version of the “Fatigue Impact Scale“ (FIS) after the acute phase of the study and after the follow-up. Secondary outcomes will cover 1) the change in the following measurements: a) functional ability, cognitive functions, and emotional symptoms subscales of the “Post-COVID-19 Symptoms Assessment Questionnaire“ (A-PASC), b) self-assessment scales of depressive and anxiety symptoms (PHQ-9; GAD-7), c) cognitive testing focused on attention, working memory, and psychomotor pace, d) quality of life monitoring based on the AQOL-6D questionnaire after the acute phase of the study and after the follow-up, and 2) evaluation of tDCS side effects during tDCS treatment. The secondary outcomes will target the electrophysiological (EEG) parameters associated with PASC and their changes after the intervention with respect to the assigned group and response rate (specifically qEEG cordance, EEG microstates, and functional connectivity will be evaluated).

Duration of the study: 04/2022 – 12/2023

Methodology:

Study design: Randomized control trial with active (n=17) and sham (n=17) group.

Subjects: 34 patients will be included in the study. Patients will be on stable medication for at least four weeks at the time of study entry. Patients will be recruited from inpatients and outpatients of Národní ústav duševního zdraví (NUDZ).

Inclusion criteria: 1. Male and female outpatients aged 18-75 years with a history of COVID-19 and monitored by an outpatient physician for mental disorders (anxiety, mood disorders, sleep) within the PASC; 2. PCR RNA SARS-CoV-2 negativity at screening/study entry; 3. Duration of symptoms >1 and ≤24 months after detection of COVID-19; 4. The mental ability to understand and sign the Informed Consent Form; 5. Presence of neuropsychiatric PASC symptoms as determined by A-PASC with a minimum overall score ≥25 (cognitive score, emotional and functional impairment); 6. FIS questionnaire score ≥40; 7. Psychopharmacological medication (if used) at a stable dose ≥4 weeks.

Exclusion criteria: 1. Contraindications of tDCS (skin disease, superficial injury, and fracture or infraction of skull in the stimulation area, epilepsy, metallic plates in the head); 2. History of any other DSM-IV axis I diagnosis prior to COVID-19, except for: 2.1. Depressive disorders, anxiety disorders, and sleep disorders that may be present in the history, but with at least 6 months of documented remission of symptoms, 2.2. Disorders associated with the use of addictive substances at least 6 months before entering the study; 3. Pregnancy or breastfeeding; 4. Patients with severe and/or unstable somatic disorders (cardiovascular disease, neoplasms, endocrinology disorders, etc); 5. Patients suffering from a serious neurological disorder (eg epilepsy, head injury with loss of consciousness)

Treatment trial:

Patients included in the study will be examined by FIS, A-PASC, AQoL, PHQ, GAD-7, and Clinical Global Impression (CGI) questionnaires to determine neuropsychiatric symptoms and the current symptom severity at study entry, at initiation, during (after 2 weeks), at the end of 4 weeks of tDCS treatment and with an interval of 4 weeks after. Attention-oriented cognitive tests (computer variant) (Digit Span - forward span), working memory (Digit Span - backwards span) and psychomotor tempo (Digit Symbol Substitution Test) will be performed at the beginning and subsequent follow-ups. In addition, three EEG examinations will be performed during the study (before the start of tDCS, after two weeks of tDCS, and at the end of tDCS treatment).
Patients will be randomly allocated according to permuted block design to one of two intervention groups: active anodal tDCS or placebo anodal tDCS.
Each tDCS session (active or placebo) will last 30 minutes. A total of 20 tDCS sessions will be administered to patients over four weeks.
The HDCStim programmable stimulator (Newronika, Italy) available for a double-blind design will be used for the application of tDCS.
Statistical analysis plan: Efficacy and safety data will be obtained from the modified intention-to-treat (mITT) dataset, consisting of patients randomized to treatment and receiving at least one tDCS session. To estimate changes in the primary outcome measurement, the Fatigue Impact Scale (FIS) total score, from baseline to week four and follow-up, a mixed model for repeated measures (MMRM) with a restricted maximum likelihood (REML) approach and Kenward-Roger adjustment of degrees of freedom will be employed. Least-squares (LS) means, within and between-group differences in LS means, and corresponding 95%CI will be calculated, and Sidak correction will be applied. Secondary outcomes (FIS subscales, A-PASC total score and subscales, PHQ-9, GAD-7, cognitive tests, and AQOL-6D) will be subjected to the same MMRM analysis, with covariate selection adapted to each outcome. The occurrence of side effects will compared by Fisher´s exact test.
