# Supplementary material for: Transcranial direct current stimulation in affecting neuropsychiatric symptoms of post-COVID syndrome: No change in microstates and functional connectivity
Source: PLoS One. 2026 Jun 26;21(6):e0351407. doi: 10.1371/journal.pone.0351407 (PMC13308831; doi:10.1371/journal.pone.0351407)
Supplement: S4 File — (PDF) [file pone.0351407.s004.pdf]

SYNOPSE

Name of the Project: **Transcranial direct current stimulation (tDCS) as a therapeutic intervention for post-acute sequelae of SARS-CoV-2 (PASC)**

Název projektu: **Transkraniální stimulace stejnosměrným proudem (tDCS) jako léčebná intervence pro zotavení z post-akutních následků SARS-CoV-2 (PASC)**

Hlavní řešitel: MUDr. Monika Klírová, Ph.D.

Cíl studie: Hlavním cílem tohoto projektu je vyhodnotit klinickou účinnost a bezpečnost transkraniální stimulace stejnosměrným proudem (tDCS) dorsolaterální prefrontální kůry (DLPFC) při léčbě neuropsychiatrických symptomů postakutních následků infekce SARS-CoV-2 (PASC). Primárním cílem dvojitě zaslepené, randomizované, placebem kontrolované studie s čtyřtýdenním následním sledováním je porovnat účinnost (odpověď na léčbu, trvání účinku) a bezpečnost 4týdenní aktivní DLPFC-tDCS (20 aplikací tDCS s umístěním anody na F3; 2 mA, 30 min/sezení) s placebem při léčbě neuropsychiatrických symptomů PASC. Primárním výsledkem studie je změna ve validované české verzi „Fatigue Impact Scale“ (FIS) po intervenci a po sledování. Sekundární výsledky budou zahrnovat 1) změnu v následujících měřeních: a) funkční schopnosti, kognitivní funkce a emoční symptomy subškály „Post-COVID-19 Symptoms Assessment Questionnaire“ (A-PASC), b) subjektivní škály pro depresivní a úzkostné symptomy (PHQ-9; GAD-7), c) kognitivní testování zaměřené na pozornost, pracovní paměť a psychomotorické tempo, d) monitorování kvality života na základě dotazníku AQOL-6D, a 2) hodnocení vedlejších účinků tDCS během léčby tDCS. Sekundární výsledky se budou zaměřovat na elektrofyziologické (EEG) parametry spojené s PASC a jejich změny po intervenci s ohledem na skupinu a míru odezvy (konkrétně bude hodnocena qEEG kordance, EEG mikrostavy a funkční konektivita).

Trvání studie: 04/2022 – 12/2023

Metodologie:

Dizajn studie: Randomizovaná kontrolovaná studie s aktivní (n = 17) a placebo (n = 17) skupinou.

Subjekty: Do studie bude zařazeno 34 pacientů. Pacienti budou v době zařazení do studie užívat stabilní medikaci po dobu nejméně čtyř týdnů. Pacienti budou rekrutováni z hospitalizovaných a ambulantních pacientů Národního ústavu duševního zdraví (NUDZ).

Kritéria pro zařazení: 1. Muži a ženy ve věku 18–75 let s anamnézou COVID-19, kteří jsou sledováni ambulantním lékařem pro duševní poruchy (úzkost, poruchy nálady, poruchy spánku) v rámci PASC; 2. Negativní výsledek PCR RNA SARS-CoV-2 při screeningu/začátku studie; 3. Trvání příznaků >1 a ≤24 měsíců po detekci COVID-19; 4. Duševní schopnost porozumět a podepsat informovaný souhlas; 5. Přítomnost neuropsychiatrických symptomů PASC stanovených pomocí A-PASC s minimálním celkovým skóre ≥25 (kognitivní skóre, emoční a funkční postižení); 6. Skóre dotazníku FIS ≥40; 7. Psychofarmakologická medikace (pokud je používána) ve stabilní dávce ≥4 týdny.

Kritéria pro vyloučení: 1. Kontraindikace tDCS (kožní onemocnění, povrchové poranění a zlomenina nebo poranění lebky v oblasti stimulace, epilepsie, kovové destičky v hlavě); 2. Anamnéza jakékoli jiné diagnózy podle DSM-IV na ose I před COVID-19, s výjimkou: 2.1. Depresivní poruchy, úzkostné poruchy a poruchy spánku, které mohou být přítomny v anamnéze, ale s alespoň 6měsíční dokumentovanou remisi příznaků, 2.2. Poruchy spojené s užíváním návykových látek alespoň 6 měsíců před vstupem do studie; 3. Těhotenství nebo kojení; 4. Pacienti s těžkými a/nebo nestabilními somatickými poruchami (kardiovaskulární onemocnění, onkologická onemocnění, endokrinologické poruchy atd.); 5. Pacienti trpící závažnou neurologickou poruchou (např. epilepsie, poranění hlavy s ztrátou vědomí)

Intervence:
Pacienti zařazení do studie budou vyšetřeni pomocí dotazníků FIS, A-PASC, AQoL, PHQ, GAD-7 a Clinical Global Impression (CGI) za účelem stanovení neuropsychiatrických symptomů a aktuální závažnosti symptomů při vstupu do studie, na začátku, během (po 2 týdnech), na konci 4 týdnů léčby tDCS, a 4 týdny po léčbě. Kognitivní testy zaměřené na pozornost (počítačová varianta) (Digit Span – forward span), pracovní paměť (Digit Span – backwards span) a psychomotorické tempo (Digit Symbol Substitution Test) budou provedeny na začátku a při následných kontrolách. Kromě toho budou během studie provedeny tři EEG vyšetření (před zahájením tDCS, po dvou týdnech tDCS a na konci léčby tDCS).
Pacienti budou náhodně rozděleni podle permutovaného blokového designu do jedné ze dvou intervenčních skupin: aktivní anodální tDCS nebo placebo anodální tDCS.
Každé tDCS sezení (aktivní nebo placebo) bude trvat 30 minut. Pacientům bude během čtyř týdnů podáno celkem 20 tDCS sezení.
Pro aplikaci tDCS bude použit programovatelný stimulátor HDCStim (Newronika, Itálie), který je k dispozici pro dvojitě zaslepený design.
Plán statistické analýzy: Údaje o účinnosti a bezpečnosti budou získány z modifikovaného souboru dat intention-to-treat (mITT), který se skládá z pacientů randomizovaných k léčbě, kteří podstoupili alespoň jedno tDCS sezení. K odhadu změn v primárním výsledku, celkovém skóre Fatigue Impact Scale (FIS), od výchozího stavu do čtvrtého týdne a následného sledování, bude použit smíšený model pro opakovaná měření (MMRM) s přístupem omezené maximální pravděpodobnosti (REML) a Kenward-Rogerovou úpravou stupňů volnosti. Budou vypočítány průměry nejmenších čtverců (LS), rozdíly v průměrech LS uvnitř a mezi skupinami a odpovídající 95% CI a bude použita Sidakova korekce. Sekundární výsledky (subškály FIS, celkové skóre A-PASC a subškály, PHQ-9, GAD-7, kognitivní testy a AQOL-6D) budou podrobeny stejné analýze MMRM, přičemž výběr kovariátů bude přizpůsoben každému výsledku. Výskyt nežádoucích účinků bude porovnán pomocí Fisherova exaktního testu.
